# Spring Suggestions

**Published:** 1861-04

**Authors:** 


**SPRING SUGGESTIONS.**—Do not take off your winter flannel sooner than the first of May, but then change to a thinner article of the same material. They are wisest and healthiest who wear woolen flannel the whole year. Sailors wear it in all latitudes and all seasons. Arrange to have a fire kept up all day in the family room, however warm it may be out of doors, until the first of May; and in the morning and evening daily until the first of June. The editor has lived in the most malarial region in the world perhaps, and when the thermometer was a hundred and twelve at noon, a fire was regularly kindled at sunrise and sunset in his office, and sat by. Disease, malignant fever, and death reigned in every direction, and yet he had not a second's sickness. It is because a brisk fire not only creates a draft, and thus purifies a room, but so rarefies the deadly air that it is carried to the ceiling where it can not be breathed. The simple precaution of having a fire kindled in the family room at sunrise and sunset in late spring and early fall, is known by eminent names in the army and navy surgery to be the most efficient preventive of all forms of fever and ague, and spring and fall diseases; in flat, wet, warm countries, it is almost a specific against those diseases. No man would be considered sane who should keep up as hot fires in his house as the spring advances as he did in mid-winter. Food is the fuel which keeps the human house—the body—warm; hence, if as much is eaten in spring as in winter, we are kept too warm; we burn up with fever; we are oppressed; we suffer from lassitude. All nature takes a new lease of life with spring but man. It is because he alone is unwise. The brute beasts, the cow, the horse, the ox; these turn to a new diet and go out to grass, to crop every green thing; they would never come to the stable or barnyard of choice to eat the "heating," "binding" oats and corn on which they luxuriated during the winter; they eat watery food which is light and purifying. Not so with man; he continues his meats and fats, his greases and his gravies, as at Christmas. Watchful nature takes away his appetite for these, and because he does not "relish" them as he did a few weeks before, he begins to conclude that something is the matter, and measuring the amount of his health by the amount he can send down his throat, he begins to stimulate the appetite, thinks he must use some tonic, readily assents to any suggestion which includes bitters and whisky, especially the latter; in addition, he puts more mustard, and pepper, and catsup on his meats, seasons every thing more heavily, until nature has been goaded so that she will bear no more, and yields to the fatal dysentery or bilious colic, or happily relieves herself by a copious diarrhea. Does not every reader know that fever, and flux, and diarrhea are common ails of spring? But you did not know one of the two chief causes, man's gluttony, as above described! Tens of thousands of lives would be saved every spring, and an incalculable amount of human discomfort would be prevented, if early in March, or at most by the first of April, meat and grease and fried food of every description were banished from the table wholly, at least for breakfast and supper. If meat will be eaten for dinner, let it be lean; use hominy and "samp" largely, have no fries, eat but little butter; use eggs, celery, spinach, vinegar; keep the body clean, spend every hour possible in the open air, snuffing in the spring; but by every consideration of wisdom and of health, have a good fire to come to and sit by with all your garments on, for eight or ten minutes after all forms of exercise; otherwise, you will wake up next morning as stiff as a bean-pole and as "sore" as if you had been pounded in a bag, to the effect of your exercise having done you more harm than good, and concluding that work don't agree with you, however beneficial it may be to others, you take no more for weeks and months. Man is certainly the biggest mule that ever was created. For the sake of giving some general idea as to how much sedentary persons should eat in spring, particularly those who are most of the time in-doors, it may be well to name the bill of fare. At breakfast, take a single cup of weak coffee or tea, some cold bread and butter, with one or two soft-boiled eggs, and nothing else. Twice a week a bit of ham or salt-fish may be used in place of the eggs, but then no meat should be eaten for dinner that day. If there is no appetite for eggs or the salt meat, it is because nature needs nothing more than the bread and butter and the drink, and nature is wise. When there is not much inclination to eat, a baked or roasted potato, with a little salt and butter, is a good substitute for an egg or piece of ham. Substitutes for these again are found in a roasted apple or in stewed fruit or cranberry-sauce. Dinner, half a glass of cold water, cold bread and butter, and a piece of lean meat, of any sort, with baked or roasted potatoes, or some other vegetable; as dessert, stewed fruits or berries of any sort, and nothing else. Supper, a single cup of weak tea, some cold stale bread and butter, and nothing else whatever; any "relish," as it is called, whether in the shape of a bit of dried beef, or cold ham, or sauce, or preserves, or cake, is nothing less than an absolute curse. This is strong language; but such things do give millions of persons restless nights, uncomfortable awakenings, and succeeding days of unwellness in every degree, from simple fidgets to ennui, ill-nature, fretfulness, and the whole catalogue of little, mean, low traits of character, such as snappishness, fault-finding, querulousness, glooms, and the like; this is because nature does not need food for supper, does not call for it; and a plain tea-table, with nothing but bread and butter on it, repels us the moment we enter the room. The next thing is to have something which has more taste in it, which "relishes;" in other words, which tempts nature to take what she would not otherwise have done; and when once inveigled into the stomach, it must be got rid of; but no preparation has been made for it; it is as unwelcome as the appearance of a friend at dinner on a washing-day. The result is, that what has been eaten is imperfectly digested, a bad blood is made of it, and this being mixed with the good blood of the system, renders the whole mass of blood in the body imperfect and impure; and as the blood goes to every part of the system, there is not a square inch of it that is not ready for disease of some sort, those parts being most liable to attack which had suffered previous injury of any kind; those who have weak brains, for example, become "softer" still, under the charitable name of "nervousness."

